# Air Exposure Affects Physiological Responses, Innate Immunity, Apoptosis and DNA Methylation of Kuruma Shrimp, *Marsupenaeus japonicus*

**DOI:** 10.3389/fphys.2020.00223

**Published:** 2020-03-12

**Authors:** Panpan Wang, Jun Wang, Yongquan Su, Zhixin Liu, Yong Mao

**Affiliations:** ^1^State Key Laboratory of Marine Environmental Science, College of Ocean and Earth Sciences, Xiamen University, Xiamen, China; ^2^Fujian Key Laboratory of Genetics and Breeding of Marine Organisms, Xiamen University, Xiamen, China

**Keywords:** *Marsupenaeus japonicus*, air exposure, oxidative stress, non-specific immunity, apoptosis, DNA methylation

## Abstract

Air exposure stress is a common phenomenon for commercial crustacean species in aquaculture and during waterless transportation. However, the antioxidant responses to air exposure discussed in previous studies may be insufficient to present the complexities involved in this process. The comprehensive immune responses, especially considering the immune genes, cell apoptosis, and epigenetic changes, are still unknown. Accordingly, we investigated the multifaceted responses of *Marsupenaeus japonicus* to air exposure. The results showed that the expression profiles of the apoptosis genes (e.g., *IAP*, *TXNIP*, *caspase*, and *caspase-3*) and the hypoxia-related genes (e.g., *hsp70*, *hif-1*α, and *HcY*) were all dramatically induced in the hepatopancreas and gills of *M. japonicus*. Heart rates, T-AOC (total antioxidant capacity) and lactate contents showed time-dependent changes upon air exposure. Air exposure significantly induced apoptosis in hepatopancreas and gills. Compared with the control group, the apoptosis index (AI) of the 12.5 h experimental group increased significantly (*p* < 0.05) in the hepatopancreas and gills. Most individuals in the experimental group (EG, 12.5 h) had lower methylation ratios than the control group (CG). Air exposure markedly reduced the full-methylation and total-methylation ratios (31.39% for the CG and 26.46% for the EG). This study provided a comprehensive understanding of the antioxidant responses of *M. japonicus* considering its physiology, innate immunity, apoptosis, and DNA methylation levels, and provided theoretical guidance for waterless transportation.

## Introduction

The Kuruma shrimp, *Marsupenaeus japonicus*, is an important commercial species that is widely distributed in the Indo-Western Pacific (Tsoi et al., 2014). Compared to other shrimps, *M. japonicus* have numerous advantages, such as fast growth rates, good reproductive capability, and survival during waterless transportation (Duan et al., 2016a). Air exposure stress is a common phenomenon for commercial crustacean species in aquaculture and during live transport (Lorenzon et al., 2008; Fotedar and Evans, 2011; Romero et al., 2011). Upon air exposure, aquatic animals are subject to water shortage and hypoxia stress, accompanied by the generation of reactive oxygen species (ROS) (Romero et al., 2007; Paital and Chainy, 2010; Paital, 2013; Abasubong et al., 2018). Air exposure affects cellular damage and metabolic capacity for *Mytilus galloprovincialis* (Andrade et al., 2019) and causes a significant but reparable immunological response in *Litopenaeus vannamei* (Xu et al., 2019). Excessive ROS have an obvious oxidation effect on lipids, nucleic acids, and proteins that leads to an imbalance of homeostasis and physiological metabolism (Romero et al., 2011; Lennicke et al., 2015; Sies, 2017). Continuous oxidative stress triggers caspase-independent cell death and induces apoptosis or necrosis (Simon et al., 2000; Wang et al., 2012, 2019; Nathan and Cunningham-Bussel, 2013; Holze et al., 2018; Ondricek and Thomas, 2018).

Crustaceans mainly rely on non-specific innate immunity, including physical defense, and cellular and humoral immunity (Sadaaki and Bok Luel, 2005; Li and Xiang, 2013). To eliminate ROS-mediated damages, organisms protect themselves with enzymatic and non-enzymatic antioxidant defenses (Storey, 1996). The antioxidant enzyme systems mainly include superoxide dismutase (SOD), glutathione peroxidase (GPx) and catalase (CAT) (Ighodaro and Akinloye, 2018). Duan et al. (2016a) investigated the effect of air exposure on antioxidant enzyme activities of Kuruma shrimp and found that the activities of SOD and CAT increased significantly. T-AOC (total antioxidant capacity), including enzymatic and non-enzymatic antioxidants, reflects the comprehensive antioxidant capacity of an organism (Jia et al., 2011; Liu H.L. et al., 2015). Air exposure induced significant oxidative and antioxidant responses in *L. vannamei* (Xu et al., 2018). Other research has detected significant changes in antioxidant enzyme activities in *Lithodes santolla* (Schvezov et al., 2019), *Paralomis granulosa* (Romero et al., 2011), *Apostichopus japonicus* (Huo et al., 2018), *Penaeus monodon* (Duan et al., 2016b), *Babylonia areolate* (Liu et al., 2017), and *Oryzias melastigma* (Cui et al., 2019). Hypoxia exposure had significant effects on the expression profiles of apoptosis and hypoxia-related genes, such as *p53*, *Hif-1*α, *Hsp70*, *GSH-Px* (glutathione peroxidase) and *CAT* (catalase) (Xiao, 2015; Sun et al., 2016a; Cai et al., 2018; Ondricek and Thomas, 2018; Camacho-Jiménez et al., 2019). Hypoxia-inducible factors (HIFs) are recognized as master regulators of the cellular response to hypoxia stress by binding HRE (hypoxia-responsive element) (Schofield and Ratcliffe, 2004; Choudhry and Harris, 2018). The role of scHIF-1α in the regulation of the HIF signaling pathway and the immune response was confirmed in mandarin fish (*Siniperca chuatsi*) (He et al., 2019). *Cancer magister* responds to hypoxia by increasing Hc concentrations and regulating subunit compositions (Head, 2010). Heat shock protein 70 (*Hsp70*) plays a vital role in preventing protein aggregation, folding and refolding of proteins and the degradation of unstable and misfolded proteins (Daugaard et al., 2007). The thioredoxin system, including TRX, TRXR, and NADPH, plays a critical role in resisting oxidative stress, which has been suggested to have complex regulatory functions in shrimp (Garcia-Orozco et al., 2012; Lu and Holmgren, 2014; Liu P.-F. et al., 2015; Zuo et al., 2019). DNA methylation, a conservative method of epigenetic regulation in eukaryotes, regulates many biological processes such as transcription, DNA repair and cell differentiation (Zemach et al., 2010; Sahu et al., 2013; Wang et al., 2015). DNA methylation levels are usually influenced by various biotic and abiotic environmental stresses, including oxidative stress (Franco et al., 2008; Murgatroyd et al., 2009; Dowen et al., 2012).

In general, air exposure is a comprehensive stimulation to aquatic animals. However, most previous studies have mainly focused on antioxidant enzyme activities. The comprehensive immune responses, especially immune genes, cell apoptosis, and epigenetic changes, are still unknown. Our objectives in the study were to determine the effects of air exposure on the physiological responses, non-specific immunity, apoptosis and DNA methylation levels of *M. japonicus*.

## Materials and Methods

### Animals

All healthy *M. japonicus* individuals (weight: 6.25 ± 1.3 g) were obtained from an aquaculture farm in Zhangzhou (Fujian, China) and were acclimated in environmentally controlled cement pools for 2 weeks (25°C, 29‰ salinity and under continuous aeration). The filtered seawater was renewed every other day and the shrimps were fed twice daily with moderate amounts of commercial pelleted food. Shrimps were fasted for 24 h before the stress treatment.

### Air Exposure and Sampling

After the conditioning period, two hundred individuals were transferred into an air-conditioned room at 25°C, and an air-exposure challenge was performed as follows. For the air-exposure treatment, the water on the surfaces of all individuals was sucked up using gauze and all individuals were then placed in six flat-bottomed rectangular tanks (80 cm × 50 cm × 30 cm). Every 20 min, these shrimp were sprayed with seawater to keep the skin moist. During the air exposure period, the hepatopancreas, gills and muscles from nine individuals were sampled at control (unstressed), 2.5, 5, 7.5, 10, and 12.5 h after exposure. Further, we recorded the numbers of dead individuals and counted the cumulative mortality. The criterion determining death was the loss of swimming ability after resubmersion. The heart rates of six individuals were determined by direct counting for 2 min at each sample point. The hepatopancreas and gills were immediately preserved in RNAfixer (BioTeck, China) for RNA extraction. Parts of the gill and hepatopancreas were rinsed with normal saline and then immediately preserved in 4% paraformaldehyde for detecting cell apoptosis. Part of the muscle and hepatopancreas tissues were immediately frozen at −80°C for lactate content determination and methylation-sensitive amplification polymorphism (MSAP) analysis, respectively.

### Homology Cloning and Sequence Analysis

Total RNA was extracted using RNAiso Plus (TaKaRa, Japan) following the specification. The purity and integrity were assessed. First-strand cDNA was synthesized using TransScript II First-Strand cDNA SuperMix (TransGen Biotech, China) according to the manufacture’s protocol. Primers were designed based on our transcriptome unigenes and sequences of other species that have been uploaded to NCBI (Table 1). PCR amplification products were ligated into the pMD19-T simple vector and sequenced in both directions. The open reading frames (ORFs) of *IAP* (inhibitor of apoptosis) and *TXNIP* (thioredoxin interacting protein) were predicted using the ORF finder^1^. The amino acid sequence was deduced by the EMBOSS Transeq^2^. ExPASy^3^ was used to analyze the protein molecular mass and isoelectric point (pI). The protein domains were predicted by SMART software^4^. Sequence alignments and phylogenetic trees were performed using MEGA 7.0 software (Kumar et al., 2016).

**TABLE 1:** Primer sequences for qRT-PCR and MSAP analysis.

**Name**	**Primer sequence (5′–3′)**	**Name**	**Adaptor and primer sequence (5′–3′)**
IAP_F1	ATTTGGAAATACAAGGAAG	Adaptors	
IAP_R1	CAGAATACTGAGGGAGGC	HMA1	CGAGCAGGACTCATGA
IAP_F2	CCAGTGACGGTTTCTATT	HMA2	GATCATGAGTCCTGCT
IAP_R2	GTGTCATCCGACTGTAGTG	EA1	CTCGTAGACTGCGTACC
IAP_F3	CAGTAAAGCCCAGTTCCA	EA2	AATTGGTACGCAGTC
IAP_R3	CAAAAACTGCCTATGATAAAAAAAT		
TXNIP5_F	ATGGCACGGAAGCTACAAAAG	Pre-amplification	
TXNIP3_R	TTACTCAAAGGTCTTCTCCTC	HM_Y	ATCCATGAGTCCTGCTCGG
Real-time PCR		EcoR_Y	GACTGCGTACCAATTCA
IAP-qRTF	CAATACCAGCACCCAGAG		
IAP-qRTR	GCTTCAGCCAAATCACAT	Selective amplification	
TXNIP_qRTF	GTCACGGACCGCAGGCAGCAG	HM1	ATCCATGAGTCCTGCTCGGCTCT
TXNIP_qRTR	CTCACTGGCGTCTTCGGACTC	HM2	ATCCATGAGTCCTGCTCGGCAT
Hsp70_qRTF	TCATCAACGAGAGCACGAA	HM3	ATCCATGAGTCCTGCTCGGTCAC
Hsp70_qRTR	TCCGCAGTTTCCTTCATCT	HM4	ATCCATGAGTCCTGCTCGGTTC
Hif1α_qRTF	CTCGCAGGACAGGATGACT	HM5	ATCCATGAGTCCTGCTCGGCTCC
Hif1α_qRTR	GACGCACTCGCTTGAACAA	HM6	ATCCATGAGTCCTGCTCGGTAC
HcY_qRTF	ACCAACAGCGAAGTCATT	EcoR1	(FAM)GACTGCGTACCAATTCACC
HcY_qRTR	GATGTCCTCACCGAAGTAG	EcoR2	(FAM)GACTGCGTACCAATTCACG
Caspase_qRTF	TTCCGGAAAACCCTCAAAC	EcoR3	(FAM)GACTGCGTACCAATTCAAC
Caspase_qRTR	GGTTTGTGTGCTTTCGGAA	EcoR4	(FAM)GACTGCGTACCAATTCATC
Caspase3_qRTF	ATTGTATTTCATTCAGGCGTGC	EcoR5	(FAM)GACTGCGTACCAATTCAGT
Caspase3_qRTR	TTGGAGGGGGATAACATAGTCTT	EcoR6	(FAM)GACTGCGTACCAATTCAGA
EF1α_qRTF	GGAACTGGAGGCAGGACC		
EF1α_qRTR	AGCCACCGTTTGCTTCAT		

### The Quantitative Real-Time PCR Analysis

The expression profiles of apoptosis and hypoxia-related genes in the hepatopancreas and gills were measured by qRT-PCR, which was implemented on the QuantStudio 6 Flex Real-time PCR System (Applied Biosystems, United States) using SYBR^®^ Premix ExTaq II (2×) (TaKaRa, Japan) following the protocols. All samples were run in triplicate and the *M. japonicus* elongation factor 1-α (*EF1-*α) was served as the reference gene (Table 1). Gene expression change was calculated by the 2^–ΔΔ^*^*C*^*^*t*^ method (Livak and Schmittgen, 2001). Expression data were shown with the mean ± standard deviation.

### Total Antioxidant Capacity (T-AOC) and Lactate Content Determination

The total antioxidant capacity (T-AOC) of the hepatopancreas tissues was determined spectrophotometrically at 520 nm according to the instructions of a commercial assay kit (Jiancheng, China). Muscle tissues were used for lactate content determination at 530 nm using a lactic acid assay kit (Jiancheng, China). The determinations were conducted in triplicate for each sample using the Infinite M200 Pro system (Tecan, Switzerland) following the protocols. One unit of T-AOC was defined as the amount of protein per mg needed to increase 0.01 of the absorbance values every minute under the assay conditions (U/mg protein). The lactate content was expressed as mmol/g protein.

### DAPI Staining and TUNEL Assay

The hepatopancreas and gills from five individuals in each group (control and 12.5 h) were sampled and preserved in 4% paraformaldehyde for 24 h. The fixed tissues were embedded in paraffin following ethanol dehydration and vitrification in dimethylbenzene. The paraffin sections were dewaxed, repaired and ruptured. The number of apoptotic cells in hepatopancreas and gills was determined using a One-Step TUNEL apoptosis assay kit (Beyotime, China) following the manufacturer’s instructions. Apoptotic cell nuclei and total cell nuclei were quantitated by TUNEL staining (green fluorescence) and DAPI staining (blue fluorescence), respectively. The double-stained sections were examined using an inverted fluorescence microscope (NIKON Eclipse TI-SR, Japan). For each sample, four visual fields were selected randomly and were photographed by an inverted fluorescence microscope. Image-Pro Plus 6.0 software was used to count the number of apoptotic and common cells. The apoptosis index (AI) was defined as the proportion of apoptotic cells in the total cells.

### DNA Methylation Levels and Patterns Under Air Exposure

Using the phenol-chloroform method, total genomic DNA was extracted from the hepatopancreas tissues of samples from the control and at 12.5 h after air exposure. The DNA integrity was visualized by agarose gel electrophoresis (1%) and concentration was measured using the Qubit 2.0 fluorometer (Life Technologies, CA, United States). DNA samples with clear bands were diluted to 100 ∼ 200 ng/μL for the downstream analysis. The MSAP analysis was performed according to Xiong et al. (1999) and He et al. (2015) with modifications. The enzyme-digestion products of *Eco*RI/*Hpa*II and *Eco*RI/*Msp*I were ligated to adapters with T4 DNA ligase (TaKaRa) and performed pre-amplification and selective amplification with fluorescent-labeled primers (Table 1).

The selective amplification products were detected by capillary electrophoresis (CE) on an ABI 3730XL auto DNA sequencer (Shanghai Sangon, China). The MSAP fragments were analyzed using GeneMapper v3.2 software. Different enzyme digestion combinations of the same DNA sample were denoted as EH/EM. Three kinds of bands were detected and counted, including EH + /EM + (type I), EH + /EM- (type II) and EH-/EM + (type III). The methylation ratio was calculated as the following formula: Hemi-methylation ratio = Type II/(Type I + II + III) and Full-methylation ratio = Type III/(Type I + II + III).

### Statistical Analysis

The data were analyzed using SPSS Statistics version 22 (IBM, United States). The results were presented as the means ± SD. The significant difference levels were detected by one-way analysis of variance (ANOVA) followed by Tukey’s test, and a *p*-value less than 0.05 (<0.05) is statistically significant.

## Results

### Sequence Analysis of *TXNIP* and *IAP*

**FIGURE 1:**
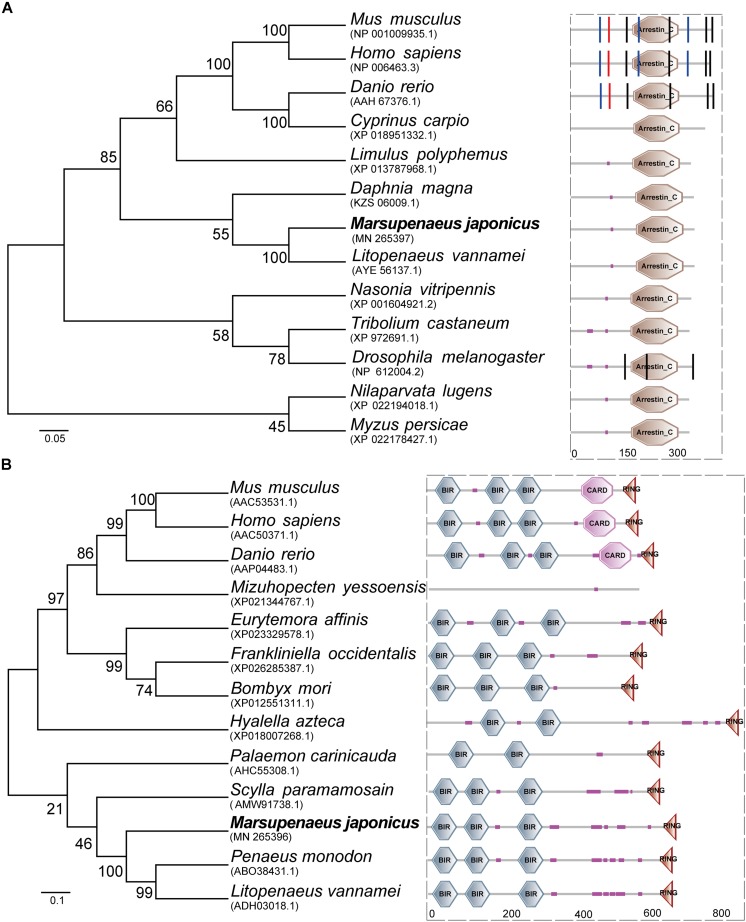
Amino acid sequence analysis of *Marsupenaeus japonicus TXNIP* and *IAP*. **(A)** Neighbor joining phylogenetic tree and protein domains analysis of TXNIPs. *Homo sapiens* (NP_006463.3), *Danio rerio* (AAH67376.1), *Drosophila melanogaster* (NP_612004.2), *Cyprinus carpio* (XP_018951332.1), *Litopenaeus vannamei* (AYE56137.1), *Limulus polyphemus* (XP_013787968.1), *Tribolium castaneum* (XP_972691.1), *Nasonia vitripennis* (XP_001604921.2), *Myzus persicae* (XP_022178427.1), *Mus musculus* (NP_001009935.1), *Daphnia magna* (KZS06009.1), *Drosophila melanogaster* (NP_612004.2). **(B)** Neighbor joining phylogenetic tree and protein domains analysis of IAPs. *Mus musculus* (AAC53531.1), *Homo sapiens* (AAC50371.1), *Danio rerio* (AAP04483.1), *Bombyx mori* (XP_012551311.1), *Eurytemora affinis* (XP_023329578.1), *Frankliniella occidentalis* (XP_026285387.1), *Hyalella azteca* (XP_018007268.1), *Mizuhopecten yessoensis* (XP_021344767.1), *Palaemon carinicauda* (AHC55308.1), *Scylla paramamosain* (AMW91738.1), *Penaeus monodon* (ABO38431.1), *Litopenaeus vannamei* (ADH03018.1).

The open reading frame (ORF) of MjTXNIP was 1044 bp (GenBank accession number: MN265397), encoding a polypeptide of 347 amino-acid residues with a calculated molecular weight of 39.97 kDa and a theoretical pI of 8.61. SMART analysis revealed that the predicted *TXNIP* protein contained an arrestin C domain located at 184-315 residues with an expected value of 2.44e-23 (Figure 1A). Homology comparisons showed that *TXNIP* shared amino acid identities with *TXNIP* from *L. vannamei* (98.85%), *Daphnia magna* (75.43%), *Limulus polyphemus* (65.99%), *Drosophila melanogaster* (61.73%), *Danio rerio* (22.67%), and *Homo sapiens* (21.52%). The open reading frame (ORF) of MjIAP (GenBank accession number: MN265396) was 2136 bp, encoding a polypeptide of 711 amino-acid residues with a calculated molecular weight of 78.48 kDa and a theoretical pI of 5.71. The SMART analysis revealed that the predicted *IAP* protein contains three BIR domains and a RING domain, which are located at 17-82, 108-172, 257-322, and 664-699 residues, respectively (Figure 1B). Multiple sequence alignment showed that MjIAP shared high amino-acid identity with *IAP* from *P. monodon* (85.96%, ABO38431.1), *L. vannamei* (84.83%, ADH03018.1) and *Scylla paramamosain* (59.73%, AMW91738.1). Phylogenetic analysis showed that the MjIAP clustered with the *IAP* protein of other crustaceans with a bootstrapping value of 100.

### Expression Profiles of *MjIAP*, *MjTXNIP*, *hsp70*, *hif-1*α, *HcY*, *caspase*, and *caspase3* Under Air Exposure Stress

**FIGURE 2:**
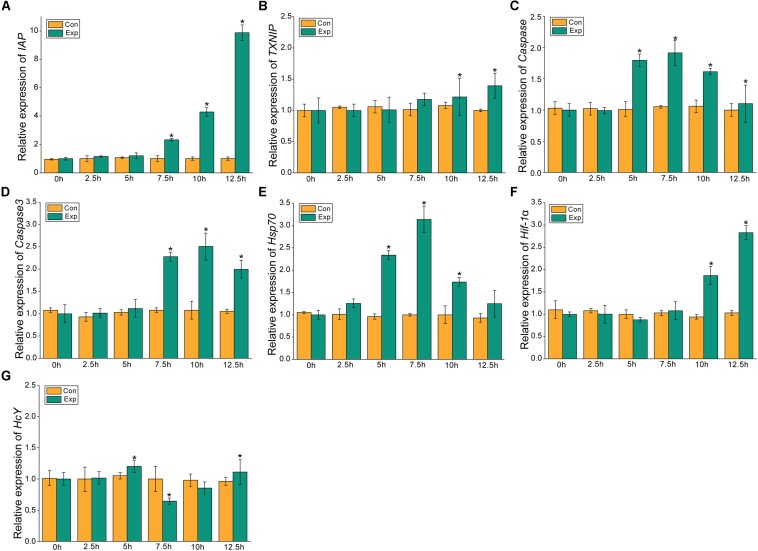
Expression profiles of several apoptosis and hypoxia-related genes in the hepatopancreas. The relative mRNA expression levels of IAP **(A)**, TXNIP **(B)**, Caspase **(C)**, Caspase3 **(D)**, Hsp70 **(E)**, Hif-1α **(F)**, HcY **(G)**. Each bar represents the mean ± SD (*n* = 6). A significant difference between groups at *p* < 0.05 (ANOVA) is indicated by “*”.

**FIGURE 3:**
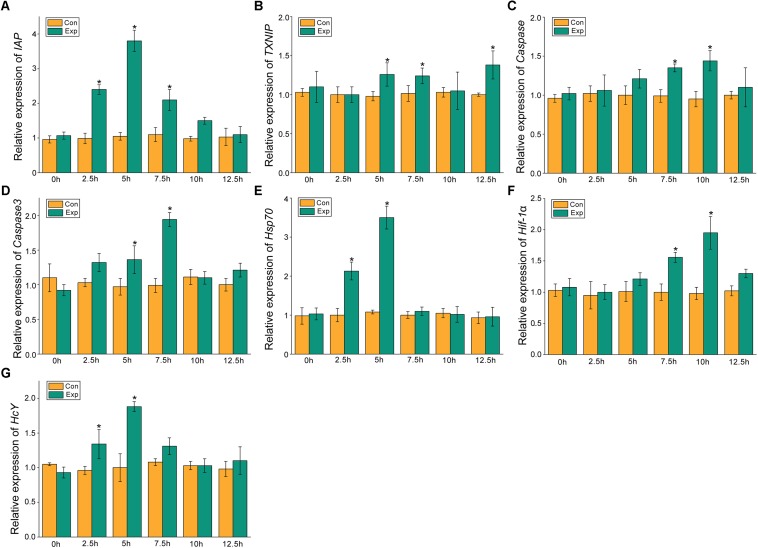
Expression profiles of several apoptosis and hypoxia-related genes in the gill. The relative mRNA expression levels of IAP **(A)**, TXNIP **(B)**, Caspase **(C)**, Caspase3 **(D)**, Hsp70 **(E)**, Hif-1α **(F)**, HcY **(G)**. Each bar represents the mean ± SD (*n* = 6). A significant difference between groups at *p* < 0.05 (ANOVA) is indicated by “*”.

The expression profiles of the apoptosis- and the hypoxia-related genes are shown in Figures 2, 3. Overall, these genes showed time-dependent changes after 5 h post-exposure in the hepatopancreas and after 2.5 h in the gill tissues. The mRNA expression levels of MjIAP gradually increased with time during air exposure. The expression levels in the hepatopancreas showed 2. 34-, 4. 3-, and 9.88-fold increases (*p* < 0.05) at 7.5, 10, and 12.5 h post-exposure, respectively (Figure 2A). In the gills, the mRNA expression levels of MjIAP showed 2.4- and 3.8-fold increases (*p* < 0.05) at 2.5 and 5 h, respectively (Figure 3A). In the hepatopancreas, the transcriptional level of MjTXNIP at the 10 h time point showed a significant incremental change (*p* < 0.05) (Figure 2B), while the expression levels in the gills peaked at 12.5 h with a 1.38-fold increase (*p* < 0.05) (Figure 3B). In the hepatopancreas, the mRNA expression levels of *caspase* increased 1.79-fold at the 5 h time point (*p* < 0.05) and peaked at the 7.5 h time point with a 1.91-fold increase (Figure 2C). In the gills, the mRNA levels showed a significant incremental change at the 10 h time point with a 1.44-fold increase (Figure 3C). The *caspase-3* levels in the hepatopancreas were significantly up-regulated at 7.5 h post-exposure and peaked at 10 h post-exposure with a 2.51-fold increase (Figure 2D). In the gills, the expression levels were significantly elevated at 5 and 7.5 h with 1.36-fold and 1.94-fold increases, respectively (Figure 3D).

After 5 h post-exposure, the *hsp70* transcriptional levels in the hepatopancreas showed significant up-regulation that peaked at the 7.5 h time point, and was 3.14-fold higher when compared with the control group (*p* < 0.05), these levels then decreased significantly back to the baseline levels (Figure 2E). In the gills, the mRNA expression of *hsp70* was significantly elevated at 2.5 and 5 h with 2.13- and 3.5-fold increases (*p* < 0.05), respectively (Figure 3E). The transcriptional levels of *hif-1*α in the hepatopancreas increased significantly by 1.87- and 2.83-fold (*p* < 0.05) at 10 and 12.5 h post- exposure, respectively (Figure 2F). In the gills, the *hif-1*α transcriptional levels showed a significant incremental change at the 10 h time point with a 1.95-fold increase (*p* < 0.05) (Figure 3F). The obvious up-regulation of the *HcY* expression in the hepatopancreas was detected at the 5 h time point with a 1.2-fold increase (Figure 2G). The *HcY* expression levels in the gills were significantly elevated at 2.5 and 5 h with 1.34- and 1.88-fold increases (*p* < 0.05), respectively (Figure 3G).

### The Cumulative Mortality and Physiological Indexes

**FIGURE 4:**
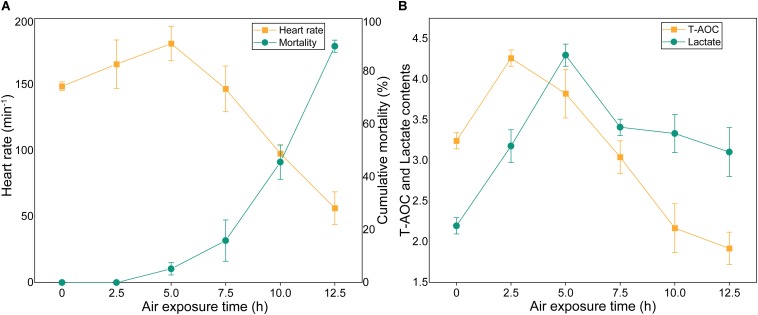
Cumulative mortality rates and physiological indexes of *M. japonicus* under air exposure. **(A)** Cumulative mortality rate and heart rates. **(B)** T-AOC and lactate contents.

The cumulative mortality and physiological indexes showed time-dependent changes upon air exposure (Figure 4). Several individuals showed stress symptoms, and nine individuals died at the 5 h time point (the mortality rate was 4.5%) (Figure 4A). As the exposure time progressed, the cumulative mortality rate was 15% at 7.5 h post-exposure, and then increased markedly to 89.5% at 12.5 h. The heart rates exhibited an initial increase and a subsequent decrease (Figure 4A). The heart rates significantly up-regulated at the 2.5 h time point and then peaked at the 5 h time point at 181 beats/min. The heart rates then quickly decreased until faint beating was observed at the 12.5 h time point. T-AOC significantly increased at 2.5 h post-exposure, which was a 1.3-fold increase (*p* < 0.05) compared with the control group (Figure 4B). After the peak, T-AOC decreased rapidly to the lowest level at the 12.5 h time point (1.9 U/mg protein). The lactate concentrations remained elevated through the 2.5 and 5 h post-exposure points, which were increased 1.72- and 2.1-fold, respectively, compared with the control group and then decreased rapidly (Figure 4B). At the 12.5 h time point, the lactate concentrations were still higher than those of the control group.

### Detection of Apoptosis in the Hepatopancreas and Gills via TUNEL

**FIGURE 5:**
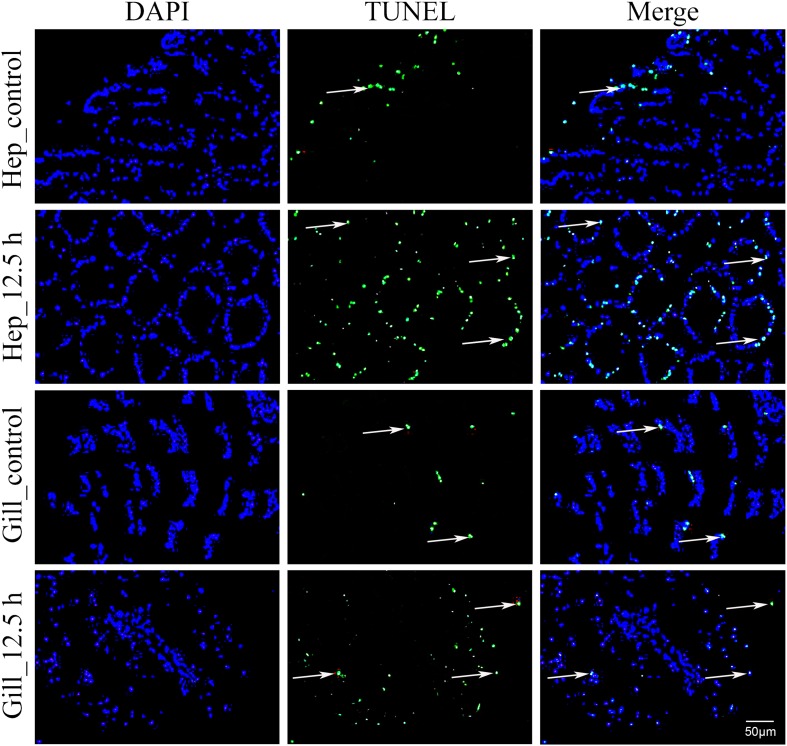
Apoptosis analysis of *M. japonicus* hepatopancreas and gill (200×). TUNEL-positive cell nuclei appear green, and parts are indicated by the arrows.

In this study, air exposure significantly induced apoptosis in the hepatopancreas and gill cells (Figure 5). TUNEL-positive cell nuclei appeared green and common cells appeared blue. For each sample, four visual fields were randomly selected for counting the apoptosis index (AI). In the hepatopancreas, the apoptosis index (AI) values of the control and the 12.5 h post-exposure group were 10.5 and 30.5%, respectively. Compared with the control group, the AI for the 12.5 h experimental group increased significantly (*p* < 0.05). In the gills, the AI for 12.5 h post-exposure group was significantly higher than that for the control group (*p* < 0.05), at 24.5 and 9.5%, respectively. Compared with the gill tissues, the hepatopancreas showed a higher mean AI at 12.5 h post-exposure. However, no significant changes (*p* > 0.05) were observed between the two groups.

### Changes in DNA Methylation Patterns in the Hepatopancreas

**FIGURE 6:**
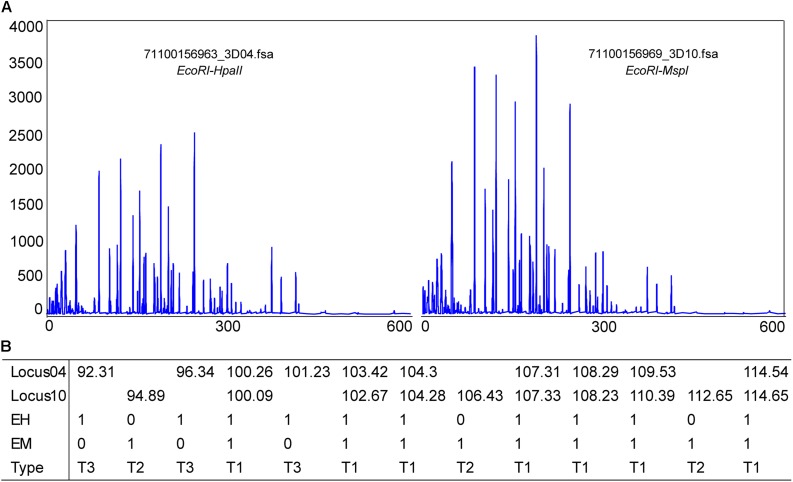
Methylation patterns detected in *M. japonicus* hepatopancreas tissues. **(A)** A chromatogram file from the GeneMapper software. The height peak indicates the quantity of amplification and the horizontal scale represents the molecular weight of the polymorphic fragments. **(B)** Polymorphic fragments were sieved and classified by size using a Python script. T1 = EH+/EM+, T2 = EH+/EM–, and T3 = EH–/EM+.

**FIGURE 7:**
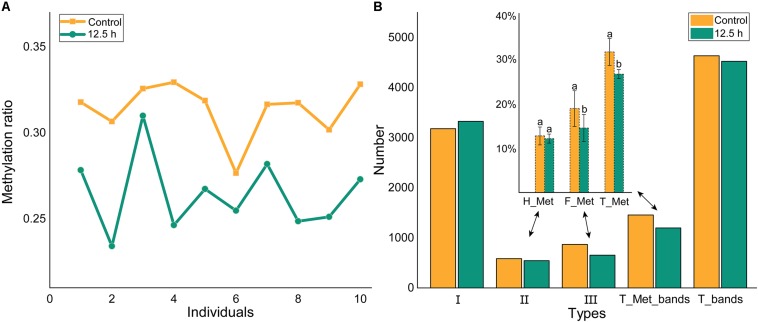
Methylation levels of individuals and groups. **(A)** The methylation ratio of ten individuals from control and 12.5 h post-exposure. **(B)** The methylation level of *M. japonicus* hepatopancreas under air exposure. I = EH+/EM+, II = EH+/EM–, III = EH–/EM+, T_Met = II + III, T_bands = I + II + III. H_Met = Half methylation, F_Met = Full methylation, T_Met = Total methylation. Each bar represents the mean ± SD (*n* = 10). A significant difference between groups at *p* < 0.05 (ANOVA) is indicated by different letters.

Methylation-sensitive amplification polymorphism fragment gel files were transformed into chromatogram files by GeneMapper (Figure 6A and Supplementary Table S1). All fragments were sieved and classified by size using a Python script (Figure 6B). The methylation ratios of ten individuals from the control group and from 12.5 h after exposure are presented in Figure 7A. Most individuals in the control group (CG) had higher methylation ratios than those in the experimental group (EG). A total of 4635 fragments for the CG and 4524 fragments for the EG were obtained (Figure 7B). The numbers of fragments of types I, II, and III were 3180, 586, and 869 for CG and were 3327, 545, and 652 for EG, respectively. The computational results showed a significant difference in the full-methylation levels (*p* < 0.05). However, there was no significant difference between the hemi-methylation levels. For both CG and EG, the full-methylation ratios were higher than the hemi-methylation ratio (Figure 7B). The average methylation ratio was 31.39% for CG and 26.46% for EG.

## Discussion

As a swimming and burrowing species, *M. japonicus* is usually subjected to aerial exposure during the processes of packing and transportation (Hewitt and Duncan, 2001; Abe et al., 2007; Fotedar and Evans, 2011). In our study, some individuals showed stress symptoms and nine individuals died at 5 h post- exposure. The cumulative mortality rate rapidly increased after 7.5 h post-exposure. At the 12.5 h time point, the remaining individuals were only sufficient for sampling. The aerial stress tolerance was consistent with the results of Duan et al. (2016a). Heart rate, a direct measure of the physiological state, is correlated with oxygen consumption and the metabolic rate (Green, 2011; Claireaux and Chabot, 2016; Lund et al., 2017). In the early stages, the heart rate significantly increased for oxygen supply and ATP production. Because the almost transparent heart is covered with a carapace, there are still no reliable tools to record heart rates. The lower heart rates were a sign of a decline in overall bodily function. During the experiment, we monitored the gill ventilation frequency, which reflected individual vitality levels. The gill ventilation frequencies exhibited an initial increase and a subsequent decrease, and could not be observed directly at the final stage.

During air exposure, T-AOC showed time-dependent changes. In the early stages of exposure, the antioxidant defense system increased T-AOC. As the exposure time progressed, the increased activity of the antioxidant enzymes was not sufficient to effectively remove ROS, however, the body needs to constantly consume antioxidants to reduce oxidative damage. Liu H.L. et al. (2015) showed that T-AOC in the hepatopancreas of *L. vannamei* decreased after 10 min of air exposure and then increased. During hypoxia, crustaceans utilize anaerobic glycolysis to satisfy their energy requirements, thus accumulating lactate (Soñanez-Organis et al., 2010; Dunbar et al., 2017; Valère-Rivet et al., 2019). For the early stages of air exposure, the lactate concentrations remained elevated through 2.5 and 5 h post-exposure, which were consistent with the results of Abe et al. (Abe et al., 2007; Aparicio-Simón et al., 2018). Subsequently, the comprehensive metabolic system of the shrimp was severely affected, and part of the lactic acid was converted into glucose to maintain homeostasis and the energy supply, thus reducing the lactic acid content.

The duration of air exposure significantly influenced the expression profiles of the hypoxia-related genes in the hepatopancreas and gills. Hepatopancreas, a multifunctional organ, integrate metabolism and immune functions. Gills, a crustacean respiratory organ, participate in ion transport, acid-base balance, ammonia excretion, and heavy metal accumulation (Henry et al., 2012). Overall, gill tissues may be more sensitive to desiccation stress than the hepatopancreas tissues, which makes sense given their direct exposure to the environment. In this study, the *Hif-1*α expression levels in the hepatopancreas and gill tissues significantly increased at the 10 and 7.5 h time points, which is in accordance with most research results (Sun et al., 2016b; Okamura et al., 2018). In the hepatopancreas, the transcriptional level of *Hif-1*α peaked at the 12.5 h time point, while the expression levels in the gills exhibited an initial increase and a subsequent decrease. As an important organ of respiration, the microvascular cavity at the edge of the gill filaments lobule is filled with a large amount of hemocyanin for oxygen transport. The *HcY* expression increased gradually within 2.5 h of air exposure in the gills, but was not obvious in the hepatopancreas. With a change in dissolved oxygen, crustaceans increase the hemolymph pH or change the composition of the Hc molecules to enhance the hemocyanin oxygen affinity (Coates and Nairn, 2014). At the initial stage of air exposure, the up-regulation of lactate concentrations might affect pH values, which weakened the binding capacity between hemocyanin and oxygen (Whiteley et al., 1997). Further studies are needed to investigate hemocyanin concentrations during air exposure.

Continuous oxidative stress triggers caspase-independent cell death and induces apoptosis or necrosis (Simon et al., 2000). Several stress conditions, such as high temperatures, hypoxia exposure, and ultraviolet (UV) irradiation, have induced extensive apoptosis in the larval embryos of the Japanese flounder (Yabu et al., 2003; Williams et al., 2017). In this study, air exposure significantly affected the percentage of TUNEL-positive cells. Compared with the control group, the apoptosis index (AI) for the 12.5 h experimental group increased significantly (*p* < 0.05) in the hepatopancreas and gills. A higher apoptosis index was detected in the hepatopancreas than in the gills, which was possibly because to the hepatopancreas is the major metabolic center of ROSs production in crustaceans (Arun and Subramanian, 1998; Moore et al., 2007). The MjIAP showed different expression patterns in the hepatopancreas and gills. An earlier upward trend was presented in gills at 2.5 h, which might suppress the early apoptosis of the gill cells. Further studies are needed to investigate the histological symptoms and ultrastructure of the hepatopancreas and gills.

As the major factors for apoptosis, the caspases are a family of structurally related cysteine proteases and contain inflammatory mediator (1,4,5,11), activator (2,8,9,10) and executioner (3,6,7) subfamilies (Shalini et al., 2015; Julien and Wells, 2017). Chronic exposure of croaker to hypoxia increased caspase-3/7 activity, as well as the apoptosis of ovarian follicle cells (Ondricek and Thomas, 2018). Hypoxia ischemia can result in a significant increase in the *caspas*e-3 expression levels and neuronal apoptosis in the brains of neonatal mice (Deng et al., 2019). In this study, the *caspase* and *caspase-3* expression levels increased initially and then decreased, and the *caspase* gene was more sensitive than the *caspase-3* gene. The *caspase* gene showed sequence similarity to the *caspase-8* gene of other species (Wang et al., 2008). The caspase protein was essential for virus-induced apoptosis in *M. japonicus*. As an activator, *caspase-8* activation can induce direct cleavage of downstream caspase-3/7 (Kaufmann et al., 2012; Feltham et al., 2017). Caspase-3/8 activities will be detected in further studies. As an endogenous inhibitor of caspases, *IAP* does not bind to *caspase-8* but inhibits its substrate, *caspase-3*, to execute anti-apoptotic roles (Wei et al., 2008). The MjIAP expression levels in the hepatopancreas gradually increased, reaching an increase of 9.88-fold (*p* < 0.05) at 12.5 h post- exposure, which may induce a decrease in *caspase* and *caspase-3* expression in the final stage. *TXNIP*, an inhibitor of the major ROS scavenger *TRX*, was positively associated with antibacterial responses but was negatively correlated with antiviral responses in shrimp (Zuo et al., 2019). In the hepatopancreas and gills, the transcriptional level of MjTXNIP exhibited similar variation trends.

DNA methylation is an important epigenetic modification, which may contribute to environmentally induced phenotypic variations by regulating gene transcription (Angers et al., 2010; Roberts and Gavery, 2012). Zhang et al. (2017) showed that the total methylation levels of *Crassostrea gigas* gills and adductors significantly increased at 12 h post-exposure. The methylation levels were different for different tissues of the same individual or for different states of the same tissue, even in the offspring of full-sibling families. The high methylation diversity among individuals may be related to the high genome heterozygosity of shrimp (Yuan et al., 2018; Zhang et al., 2019). The control group and the experimental group all had one abnormal individual, but this did not affect the overall outcome. The air exposure led to a lower methylation ratio in the experimental group than in the control group. There are varying degrees of methylation in invertebrates, which are often below the rate for vertebrates (Gavery and Roberts, 2010; Roberts and Gavery, 2012). The methylation rates in this study were higher than those of *Fenneropenaeus chinensis* (He et al., 2015). DNA methylation has an obvious tissue-specific pattern (Sun et al., 2014; Zhao et al., 2015). Except for the hepatopancreas, the DNA methylation changes in other tissues still need further verification. It is well known that DNA methylation in promoter regions normally represses gene expression (Suzuki and Bird, 2008; Maunakea et al., 2010; Jones, 2012; Zhou et al., 2019). Wan et al. (2015) determined the existence of two different modes of DNA methylation-dependent gene regulation. The DNA methylation of *IAP*, *TXNIP*, *caspase*, and *caspase-3* genes may play a key role in the differential expression patterns, which needs further verification.

## Conclusion

This study provided a relatively comprehensive understanding of the desiccation and oxidative stress responses of *M. japonicus* in terms of physiology, innate immunity, cell apoptosis, and DNA methylation levels, and facilitated further investigation into the molecular mechanisms of shrimp under oxidative stress. In the coming centuries, aquatic animals will face more severe challenges, including ocean warming and the expansion of the death zone (Keeling et al., 2010; Altieri and Gedan, 2015; Vinagre et al., 2019). This research can serve as a case study for future research on hypoxia adaptation and the antioxidant mechanisms of other marine groups.

## Data Availability Statement

The data of MjTXNIP (MN265397) and MjIAP (MN265396) are publicly accessible in the NCBI (https://www.ncbi.nlm.nih.gov/).

## Ethics Statement

All experimental procedures were conducted in conformity with institutional guidelines for the care and use of laboratory animals in Xiamen University, Xiamen, China.

## Author Contributions

PW, YM, JW, and YS designed the experiments. PW and ZL conducted computational and statistical analyses. PW and YM drafted the manuscript. All authors read and approved the final manuscript.

## Conflict of Interest

The authors declare that the research was conducted in the absence of any commercial or financial relationships that could be construed as a potential conflict of interest.

## Footnotes

1 https://www.ncbi.nlm.nih.gov/orffinder/

2 https://www.ebi.ac.uk/Tools/st/emboss_transeq/

3 https://www.expasy.org/

4 http://smart.embl.de/
